# Palladium-Based Contacts on p-GaN and Their Application in Laser Diodes

**DOI:** 10.3390/ma16196568

**Published:** 2023-10-06

**Authors:** Iryna Levchenko, Serhii Kryvyi, Eliana Kamińska, Szymon Grzanka, Ewa Grzanka, Łucja Marona, Piotr Perlin

**Affiliations:** 1 Institute of High Pressures Physics, Polish Academy of Sciences, 01-142 Warsaw, Poland; ekaminska@unipress.waw.pl (E.K.); szgrzanka@unipress.waw.pl (S.G.); ewa.grzanka@unipress.waw.pl (E.G.); lucja@unipress.waw.pl (Ł.M.); piotr@unipress.waw.pl (P.P.); 2 Institute of Physics, Polish Academy of Sciences, 02-668 Warsaw, Poland; kryvyi@ifpan.edu.pl; 3 Institute for Nanoscience and Engineering, University of Arkansas, Fayetteville, AR 72701, USA

**Keywords:** gallium nitride (GaN), thermal evaporation, solid-state solution, diffusion, palladium (Pd), nickel (Ni), gold (Au), ohmic contact, specific contact resistance (ρ), laser diode (LD)

## Abstract

In this paper, we investigate the effect of Pd thickness and heat treatment on Pd/Ni/Au/p-GaN metal contacts. The as-deposited samples exhibit a smooth morphology and non-linear I–V characteristics. Heat treatment in a N_2_ atmosphere leads to degradation of the contact microstructure, resulting in diffusion of Ga, void formation on the interface and mixing of metals. Annealing in a mixture of N_2_ and O_2_ improves adhesion and reduces contact resistance. However, this process also induces GaN decomposition and species mixing. The mixing of metal–Ga and metal–metal remains unaffected by the method of thermal treatment but depends on gas composition for thin Pd contacts. To achieve low-resistance contacts (≈1 × 10^−4^ Ω cm^2^), we found that increasing the Pd thickness and using N_2_ + O_2_ as the annealing environment are effective measures. Nevertheless, the degradation effect of the annealed contact microstructure in the form of the void generation becomes evident as the thickness of Pd increases. Laser diodes (LDs) with optimized palladium-based contacts operate at a voltage of 4.1 V and a current density of 3.3 kA/cm².

## 1. Introduction

Optoelectronic devices based on AlInGaN nitride semiconductors can generally be divided into light-emitting diodes, laser diodes and photodetectors, especially UV photodetectors. All these types of devices are capable of emitting and detecting light in a broad range including infrared (IR), visible and ultraviolet (UV) spectra. Historically and economically, the most important devices are light-emitting diodes, which, when combined with phosphors, form white LED light bulbs. These bulbs serve as the primary source of light for our civilization [1]. Laser diodes based on nitride semiconductors were originally developed for high-density optical storage (Blu-ray standard) [2]. They have now found applications in laser lighting [3], laser processing (copper welding) [4], sensing and quantum technologies [5]. With their high application relevance, wide-bandgap AlInGaN compounds present technological challenges, which include the formation of ohmic contacts. This is due to the low p-type conductivity and the high ionization energy of the acceptors in GaN [1]. The latter makes it difficult to form low-barrier contacts with p-type GaN as there are no metals with a high enough work function to match the high energy gap of GaN [1]. While achieving a low-resistivity ohmic contact with p-GaN poses challenges in AlInGaN LEDs, this issue becomes even more pronounced for nitride laser diodes (LDs), where the operational current density reaches kiloamperes per square centimeter (kA/cm²). The primary performance concern for laser diodes (LDs) is the significant voltage drop observed at the p-GaN–metal interface. Given that LD diodes typically operate at high current densities, the presence of a high-quality p-type ohmic contact becomes crucial in mitigating the operating voltage. Failing to address this issue could lead to excessive heat generation within the device due to the elevated current levels, consequently limiting the device’s overall lifetime, reliability and performance. The attainment of a low-resistance ohmic contact hinges on various critical factors, encompassing the treatment of the semiconductor surface, the composition of the metallization, the thickness of the contact layers and the environmental conditions during the contact manufacturing process. Ideally, the metal–semiconductor interface should be inert, devoid of oxides and defects and exhibit an atomically smooth surface along with an epitaxial metal film. In principle, the resistivity of the ohmic contact ought to be contingent on the metal’s work function, as expected given the strongly ionic nature of GaN. The characteristics of GaN, which possesses a band gap of 3.4 eV and an electron affinity ranging from 3.8 to 4.1 eV [6,7], necessitate a metal featuring a work function spanning from 7.2 to 7.5 eV to establish a barrier-free interface. Regrettably, currently accessible metals possess a work function that falls below 6 eV. Consequently, a notable energy barrier hinders the hole injection. Nevertheless, metals featuring a higher work function, when directly deposited onto GaN, have the capability to diminish the magnitude of the potential barrier at the metal–semiconductor interface. In a study referenced as [6], the authors have demonstrated that the resistance experiences an exponential decrease with the augmentation of the metal’s work function. This observation applies to metals such as Pt, Ni, Pd, Au, Cu, Ti, Al and Ta. However, adopting a multilayer contact approach based on high-work-function metals does not invariably guarantee a reduction in contact resistance on p-GaN. Furthermore, even low-resistance contacts such as Ta/Ti, Pd/Al, Ti/Al/Ni/Au, Ni/Au and Ni/C/Au can exhibit instability in ambient air both before and after annealing, primarily due to the chemical reactivity of the metal with GaN and its subsequent thermal instability. In the context of ohmic contacts, the precise control of a uniform surface morphology becomes pivotal. Different metal microstructures and orientations can result in substantial alterations to contact properties [8]. In the absence of thermal treatment, when p-GaN is moderately doped (approximately 10^17^ cm^−3^) and the GaN surface remains inactivated by chemical processes like RIE or selective etching, most metals tend to form non-ohmic contacts. Studies have been conducted to demonstrate the feasibility of achieving low-resistivity, non-annealed, ohmic contacts on p-GaN [9]. Nonetheless, concerning the fabrication processes for laser diodes (LDs), contacts that remain thermally untreated are undesirable. Complications arise from the surface dissociation activated by the metal, which takes place at relatively low temperatures during the formation of the contact. Reactions between metal and semiconductor constituents can lead to sustained deterioration of the contact over time. Additionally, excessive interaction at the interface can exert an adverse influence on device performance. It is important to highlight that, in certain instances, the newly formed compound at the interface during contact annealing may possess a higher work function and contribute to a thermally stable contact structure.

Following annealing at elevated temperatures, the semiconductor–metal interface develops a heightened density of structural defects. These defects stem from the relaxation of internal mechanical stresses, which arise due to the mismatch between the electrical and structural characteristics of the metal–semiconductor heterojunction [10]. However, thermal annealing serves to enhance adhesion while concurrently diminishing the specific contact resistivity. Occasionally, the choice of a contact scheme hinges upon the metal’s capability to generate stable compounds with GaN and facilitate near-surface doping. The introduction of a new interlayer can also heighten the likelihood of tunneling. In a previous study [11], annealed Pt/Ni/Au and Pt/Au contacts on p-GaN demonstrated commendable ohmic behavior due to interfacial reactions between Pt and p-GaN. The formation of crystalline NiO alongside the amorphous Ni–Ga–O phase during Ni/Au annealing contributed to a reduction in contact resistance for p-type GaN [12]. Previous research efforts have primarily concentrated on examining the electrical properties of contacts on p-GaN. A comprehensive examination of the metallization structure following successive processing stages and the influence of contact processing on GaN laser properties has yet to be systematically undertaken. The adept management of interfacial processes and the creation of a preferred contact structure characterized by low contact resistivity are poised to significantly contribute to the advancement of technology and foster a deeper comprehension of the metal–GaN system, ultimately enhancing device reliability.

In this study, we explore the p-GaN/Pd/Ni/Au contact system by introducing variations in thermal treatment parameters, encompassing temperature, gas environment, the type of annealing equipment employed as well as alterations in Pd thickness. The insights derived from these investigations are instrumental in comprehending the behavior of this metallization scheme on p-type GaN. They also offer strategic avenues to enhance the technology behind ohmic contacts for nitride semiconductors.

## 2. Materials and Methods

All experiments were conducted on GaN/sapphire templates. The structures, designed according to typical LD configurations, were grown using metalorganic vapor phase epitaxy (MOVPE). These structures comprised 2 µm of GaN, succeeded by 500 nm of GaN doped with a Mg concentration of 1 × 10^19^ cm^−3^ (exhibiting a free-hole concentration of 5 × 10^17^ cm^−3^) and a 10 nm GaN-p^++^ subcontact layer featuring a Mg concentration of 2 × 10^20^ cm^−3^. Prior to depositing the metal contacts, the surface of the p-GaN samples underwent cleaning with dimethyl sulfoxide (DMSO), followed by etching in aqua regia (1HNO_3_-3HCl), hydrofluoric acid (HF) and hydrochloric acid (HCl). Following the chemical treatment, Pd/Ni/Au metals with a nominal thickness of 10–90 nm/10 nm/30 nm were deposited through an e-beam evaporator operating at a vacuum pressure of 2 × 10^−7^ Torr. Pd, Ni and Au boast substantial work functions, which can result in a diminished Schottky barrier height (SBH) when interfacing with p-GaN. Furthermore, these metals possess elevated melting points and exhibit comparable coefficients of thermal expansion (as delineated in Table 1).

Gold is a metal known for its outstanding electrical and mechanical properties, coupled with a relatively elevated work function. Being a noble metal, it does not establish direct bonds with semiconductor materials, GaN included [14]. Nickel is recognized for its exceptional adhesion to a wide range of semiconductor materials, GaN being no exception [15]. Palladium, apart from its high work function, exhibits remarkable resistance to oxidation. We applied three methods of thermal treatment:

1. Rapid thermal annealing (RTA) at a temperature of 530 °C in a neutral ambient (N_2_ flow): heating for 2 min, annealing for 10 min, cooling for 3 min;
2. Furnace annealing at a temperature of 530 °C in an oxidizing atmosphere (N_2_ + O_2_ + H_2_O): preheating up to 170 °C for 2 min, heating for 20 min, annealing for 10 min, slow cooling for 10 min, air cooling;
3. Rapid thermal annealing at a temperature of 530 °C in an oxidizing atmosphere—artificial air (N_2_ + O_2_ flow): heating for 2 min, annealing for 10 min, cooling for 3 min.

After the heat treatment process, we employed several characterization methods to assess the properties of the metal contacts to p-GaN. These methods encompassed the following:

Transmission Line Method** **(TLM) was utilized to ascertain the specific contact resistance;

Scanning Electron Microscopy (SEM) was employed to visualize the surface morphology of samples subjected to different annealing approaches;

Transmission Electron Microscopy** **served to observe alterations in the metallization structure subsequent to diverse annealing procedures;

Energy-Dispersive X-ray Spectroscopy (EDX) offered insights into the composition of the Pd/Ni/Au/GaN contact system;

X-ray Diffraction (XRD) was employed for structural characterization of the samples. Measurements were conducted using the Empyrean X-ray diffractometer operating at the Cu_Kα1 wavelength, equipped with a hybrid two-bounce monochromator.

To provide a concise overview, the experimental techniques were employed in the following manner:

The specific contact resistivity was deduced by analyzing the current–voltage characteristics (I–V) of the Pd/Ni/Au contacts through the application of the TLM method. Rectangular pads were fabricated on the p-type GaN contact layer using a standard photolithography technique.

Structural examinations and evaluations of elemental composition were conducted utilizing an FEI Titan Cubed 80–300 microscope operating at 300 kV, incorporating objective lens correction.

High-resolution transmission electron microscopy (HR-TEM) images were captured using a UltraScan 1000 charge-coupled device (CCD) camera (Gatan Inc.,USA). High-resolution scanning transmission electron microscopy (HR-STEM) in annular dark-field mode employed the high-angle annular dark-field (HAADF) Fischione 3000 detector (E.A. Fischione Instrumental Inc., USA). High-resolution images were acquired for GaN $\left[11\bar{2}0\right]$ zone axis (ZA) orientation (electron beam was parallel to the $\left[11\bar{2}0\right]$ crystallographic direction). Fast Fourier transformations (FFT) of high-resolution electron microscopy images were used to define the crystallographic orientation of metal layers regarding the GaN substrate. FFT of HR-STEM images were also used to determine the particular interplanar spacing of the metal layers. For this purpose, FFT in each case was scaled according to the theoretical interplanar values of GaN. Energy-dispersive X-ray spectroscopy (EDX) maps were collected in TEM using an EDAX 30 mm^2^ Si(Li) detector with a collection angle of 0.13 srad. The integration time for EDX acquisition was set at 1 s per pixel. Thin lamellas for TEM examination were prepared using the focused ion beam (FIB) technique in the Helios Nanolab 600 (FEI, USA). A platinum protection layer was deposited to safeguard the top surface, and the thin lamellas were transferred onto a copper TEM grid.

## 3. Results and Discussion

### 3.1. As-Deposited Pd/Ni/Au Contact on p-GaN

**Figure 1:**
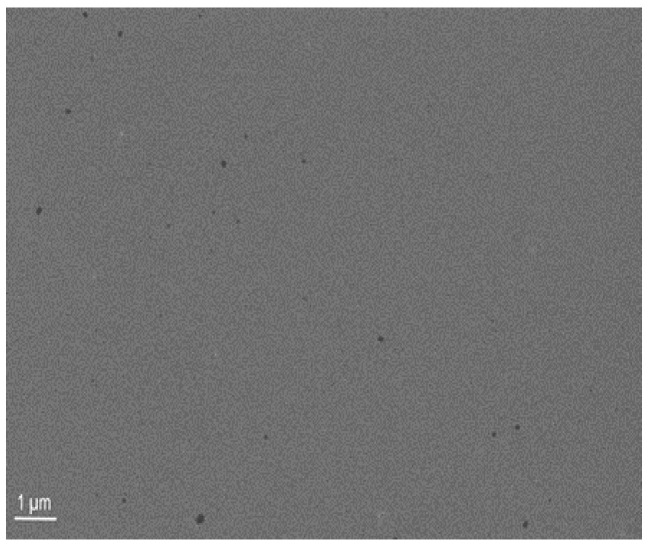
SEM image of the surface of as-deposited Pd/Ni/Au (10/10/30 nm) metallization on p-GaN. The provided layer thicknesses correspond to the settings used for evaporation.

Following the previously described wafer-cleaning procedure, we employed the e-beam evaporation technique to deposit metal layers of Pd/Ni/Au with nominal thicknesses of 10/10/30 nm. The metallization in its as-deposited state showed a consistent and even surface morphology, as depicted in Figure 1.

**Figure 2:**
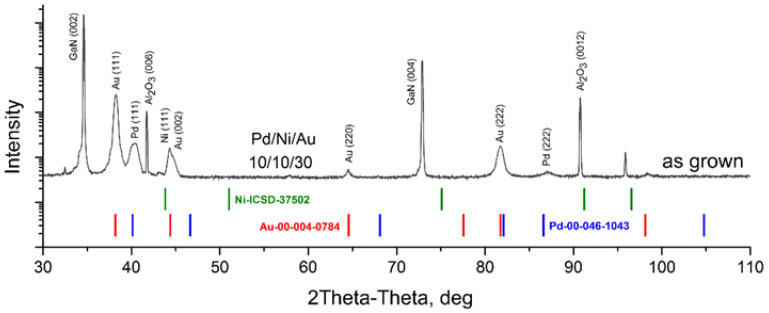
XRD profile for investigated Pd/Ni/Au (10/10/30 nm) as-grown sample. The powder diffraction patterns with corresponding reference numbers in PDF-2 and ICSD databases are shown.

For the as-deposited metallization, distinct phases of pure Au, Ni and Pd were observed, with no detectable metal intermixing in XRD measurements (Figure 2). No mixing of layers was observed.

**Figure 3:**
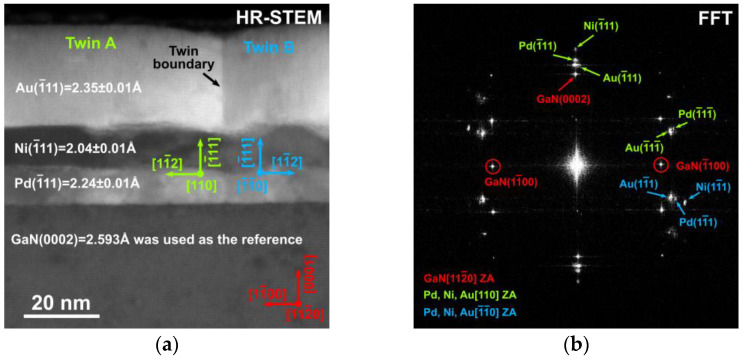
HR-STEM image (**a**) of as-deposited Pd/Ni/Au (10/10/30 nm) together with the interplanar distance for the indicated lattice planes (**b**) determined from measurements by FFT. ZA—zone axis.

Cross-sectional TEM analyses were conducted to describe the interfacial structure of the studied samples. TEM images reveal a clear demarcation between the metal and GaN layers (Figure 3a). The metal layers exhibit a preferred orientation of crystalline structure and the presence of twinned structures within the deposited metal layers is observed. The interlayer spacing determined from the Pd/Ni/Au/p-GaN structure for the (111) plane measures 2.24 Å, 2.04 Å, 2.35 Å and 2.593 Å, respectively. The HR-STEM image displays well-defined metal-layer interfaces and thicknesses that closely correspond to the structure design.

The deposited metals are highly textured and show crystallographic orientation with respect to the substrate, described as follows: ${\left[\bar{1}11\right]\;}_{Metal\;layer}∥{\left[0001\right]}_{GaN}$ in the growth direction and ${\left[110\right]\;}_{Metal\;layer}∥{\left[11\bar{2}0\right]}_{GaN}$, ${\left[112\right]\;}_{Metal\;layer}∥{\left[10\bar{1}0\right]}_{GaN}$ in lateral directions (Figure 3b).

**Figure 4:**
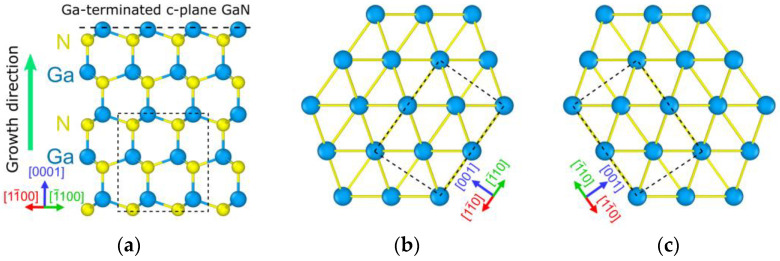
Crystallographic orientation of GaN (**a**) and metal layers (**b**,**c**).

The schematic view of the crystal lattice for GaN’s $\left[11\bar{2}0\right]$ and the metals’ $\left[110\right]$ zone axis orientations is shown in Figure 4.

The crystal lattices are aligned in accordance with the experimental TEM data. Figure 4b,c corresponds to the two observed types of twins in the TEM. The dashed rectangles correspond to the elemental crystallographic lattice for each structure.

**Figure 5:**
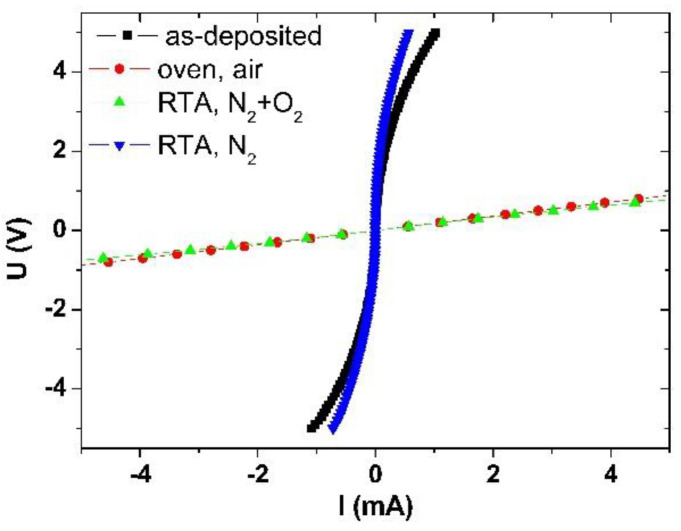
I−V plots for the as-deposited and annealed contacts.

The TLM technique was employed to characterize the contact resistivity (Figure 5).

The I–V characteristics of the as-deposited contacts showed a strong non-linearity, with a resistivity of 7 *×* 10^−1^ Ωcm^2^.

### 3.2. Pd/Ni/Au Contact on p-GaN Annealed in N_2_ Atmosphere

**Figure 6:**
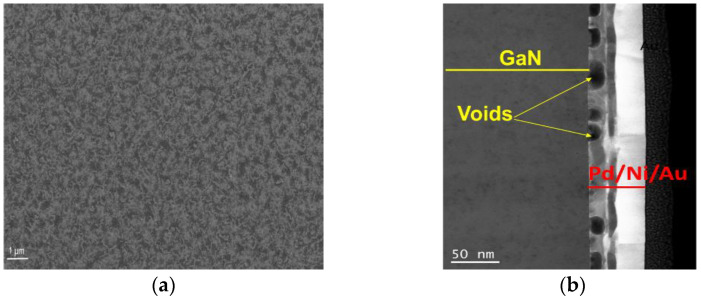
SEM (**a**) and STEM (**b**) images of Pd/Ni/Au (10/10/30 nm) annealed in RTA in N_2_ flow at 530 °C.

The SEM image (Figure 6a) of samples annealed under a nitrogen flow illustrates that the top surface of the metallization exhibits a more granular appearance compared to the as-deposited sample, while maintaining an overall smooth texture. STEM data still reveal three relatively well-defined layers, although the palladium layer displays a dense network of voids (Figure 6b).

**Figure 7:**
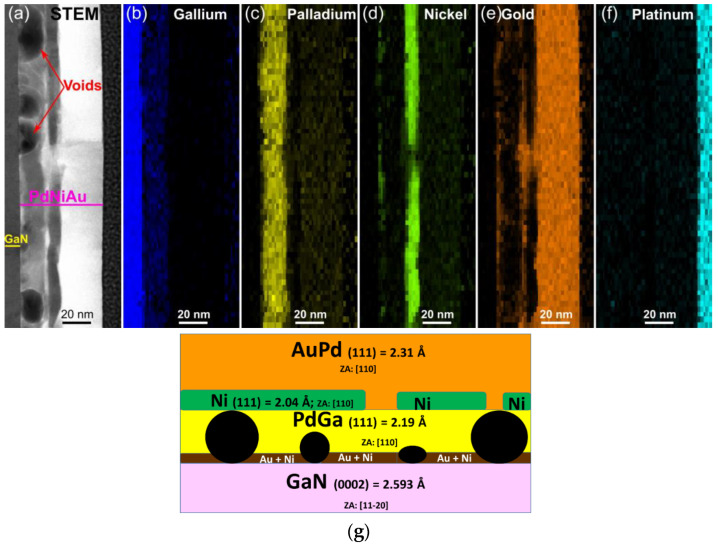
EDX elemental mapping data for p-GaN/Pd/Ni/Au (10/10/30 nm) contact annealed in N_2_ (**a**–**f**). Schematic view of the contact composition (**g**).

The EDX data demonstrate a more intricate STEM image of our annealed three-layer structure (Figure 7a–f). Annealing evidently prompts the diffusion of Ga atoms from the GaN semiconductor into the adjoining Pd layer, thereby giving rise to PdGa clusters. This infusion of Ga atoms into the Pd layer and generation of voids of varying sizes lead to an almost twofold expansion in the thickness of the latter layer.

The precise mechanism behind the formation of voids adjacent to the semiconductor–metal interface is still a subject of debate; however, we can propose two hypotheses: the de-wetting process and the nitrogen release. The first is linked to the wettability of the semiconductor’s surface (or any ceramic material). It has been demonstrated that thin metal layers deposited on diverse dielectric materials (e.g., gold on SiO_2_) can undergo a de-wetting process, associated with the interplay between surface energy and bulk cohesion [16]. De-wetting involves the transformation from a continuous metal layer into droplets on the surface because of annealing. The second plausible factor contributing to void formation could be associated with the release of nitrogen from the subsurface of the semiconductor. Nitrogen, forming N_2_ molecules [15], might introduce a pressure component into the mechanism, driving void formation. Notably, voids are distributed randomly at the interface. The existence of these voids reduces the contact area between the metal and the semiconductor by almost 60%. This phenomenon inevitably leads to an elevation in the contact resistance that corresponds to the extent of the actual contact.

The diffusion of Pd into the Au layer occurs alongside the creation of an intermetallic solid solution of Pd in Au (Figure 7g). The interaction between Pd, Au and Ga could be advantageous from an electronic structure perspective under the given thermal conditions. As highlighted in [17], pure palladium has 9.4 electrons per atom in the d-band. To fully fill the d-band of Pd (5s^0^4d^10^), an additional 0.6 electrons are necessary. The density of available d-states surpasses that of s-states. Consequently, the s electrons from both gallium (4s^2^4p^1^) and gold (5d^10^6s^1^) migrate into the d-band of palladium to complete its occupancy. The feasible bonding configurations involve either metallic Ga and Pd or covalent Ga and metallic Pd. This formation of a solid solution of Pd in Au contributes to reduced stresses due to a diminished lattice mismatch with GaN.

The thin layer of Ni-and-Au mixture situated between GaN and the metallization points to an ongoing interfacial reaction. GaN and Pd are divided by Au–Ni clusters. Unlike [18], no Ni_x_Ga_y_ or Ni_x_N_y_ phases were observed. It is well established that Ga-face p-GaN exhibits robust chemical resistance [19].

The inert behavior displayed by Ga-face material finds its explanation in the state of surface bonding. Specifically, the outer layer consists of Ga atoms, forming bonds with three N atoms at the surface and one N atom in the adjacent layer. The chemical reactivity of the GaN substrate is influenced by the lack of electron lone pairs. Notably, our experimental results illustrate instances of GaN decomposition and the subsequent diffusion of gallium atoms into the underlying Pd layer. This phenomenon could potentially be clarified by considering semiconductor activation due to the presence of dangling or defective Ga bonds within regions abundant in defects.

We propose that the annealing process at a relatively low temperature (530 *°*C) renders GaN less stable. The presence of Pd species along with Ni at the interface acts as a potent thermodynamic driving force, leading to the rupture of Ga–N bonds, decomposition into liquid Ga and subsequent nitrogen release [20].

The interfacial reaction between Pd and p-GaN induces covalent atomic interactions [21]. Based on fast Fourier transform (FFT) analysis of HR-STEM images, PdGa, Ni and AuPd species exhibit growth in the same direction toward GaN. According to [22], gallides of palladium are notably more stable compared to other group VIII metal gallides. Therefore, in our case, Pd is thermodynamically favored to interact with p-GaN.

Palladium, possessing a higher surface energy, agglomerates to minimize the total interfacial energy of the metal–semiconductor system. The thermal expansion coefficient of Pd is twice that of GaN (11.7 *×* 10*^−^*^6^ K^−1^ vs. 6 *×* 10*^−^*^6^ K^−1^). The annealing process could generate compressive stress exceeding the ultimate tensile strength of Pd, leading to layer de-wetting. The lateral non-uniformity of metallization upon annealing could negatively impact the electrical characteristics.

Furthermore, due to the constraints of the metal deposition technique employed, achieving an epitaxial metallization structure remains elusive. The evaporated metal is subjected to compressive stress [23], contributing to the emergence of nanovoids on the GaN surface, resembling ceramic characteristics. This phenomenon is observed even without subjecting the samples to thermal treatment [24]. Notably, upon annealing, the newly formed metallization composition did not exhibit the capacity to disband the nitrogen atoms or prevent void formation. Determining the potential mechanism behind the formation of these voids during contact treatment should consider the coalescence of nanovoids as a significant factor.

In a general sense, annealing through RTA within a nitrogen-rich environment serves to hinder the out-diffusion of Ni towards the upper surface of the metallization layer, subsequently preventing its oxidation by enabling its dissolution into the Au layer. Oxidation of Ni is a trait typical of Ni/Au contacts annealed in an O_2_ atmosphere. However, partial diffusion of Ni and Au to the PdGaN interface persists. Given the high diffusivity of metals, both Ni and Au species wet the GaN surface and segregate from the PdGa layer, as illustrated by the EDX maps.

**Figure 8:**
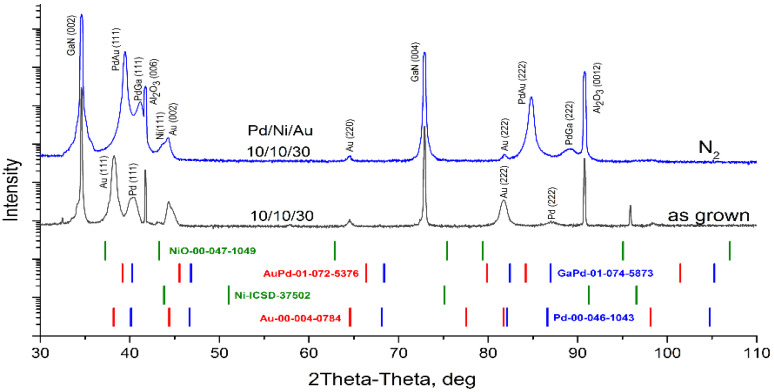
XRD profiles for investigated as-grown sample and after annealing in N_2_.

The above EDX analysis was confirmed by XRD data (Figure 8). XRD findings suggest that Pd is entirely dissolved and PdGa and PdAu phases are detected. Lowering (but not disappearance) of Au (002) and Ni (111) reflection intensity indicates that these layers have almost completely been consumed through interfacial and interlayer reactions.

Thermal treatment within the nitrogen atmosphere does not significantly enhance the contact quality. Current–voltage measurements reveal a non-linear characteristic (see Figure 4). The specific contact resistivity decreases to 3.7 × 10^−1^ Ωcm² compared to the as-deposited layer. However, due to the contact area being reduced by approximately two times, there is a substantial fourfold enhancement relative to the as-deposited contact. Nevertheless, the high contact resistance and its non-linear nature render this metallization unsuitable for practical applications. The obtained value is four times higher compared to the contact annealed at 500 *°*C in N_2_ for 1 min as reported in [25]. This discrepancy suggests that extended annealing periods may lead to the degradation of the electrical properties of Pd/Ni/Au metallization. On the contrary, it *is* important to consider that such a short annealing time may not be sufficient for the stabilization of the contact structure.

### 3.3. Pd/Ni/Au Contact on p-GaN Annealed in Oxidizing Atmosphere

**Figure 9:**
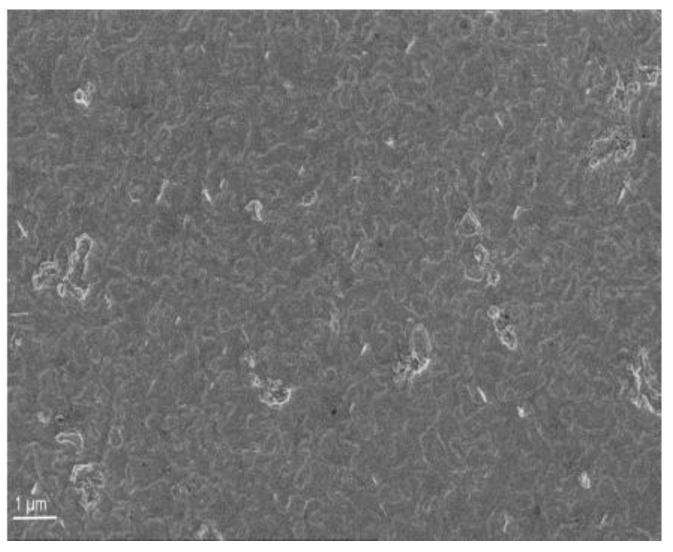
SEM data of Pd/Ni/Au (10/10/30 nm) annealed in RTA in N_2_ + O_2_ flow at 530 °C.

Annealing in an oxygen-containing environment was historically a pivotal step in producing semi-transparent, low-resistance Ni/Au contacts for early InGaN-based LEDs [12]. Recognizing the critical impact of oxygen during the annealing process for Ni-containing metallization, we chose to follow this route for our palladium–nickel-based contacts. Drawing from our prior experience with oxidized Ni/Au contacts, we anticipate significant reactivity of species in this scenario as well. Our Pd/Ni/Au metallization underwent annealing in a mixture of N_2_ and O_2_ or in ambient air at 530 °C. Figure 9 depicts the rugged surface morphology and microstructure of the metallization post-annealing. Remarkably, the morphology of the annealed Pd/Ni/Au contact closely resembles that of a standard oxidized Ni/Au contact.

**Figure 10:**
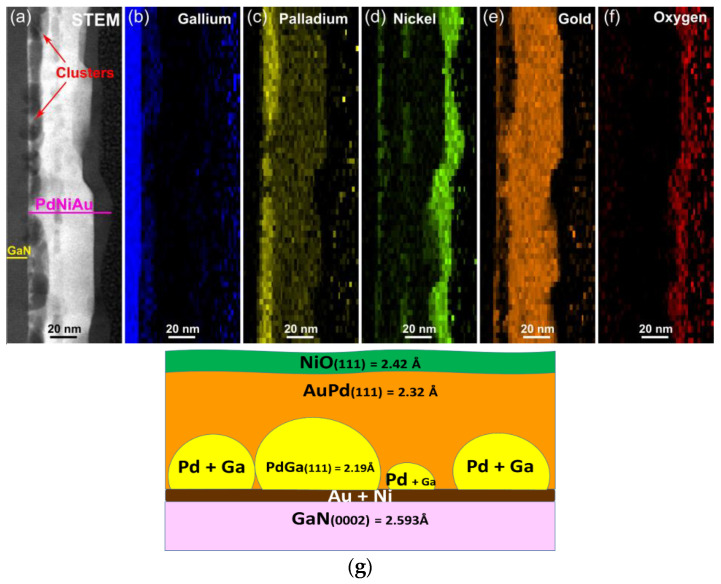
EDX elemental mapping data (**a**–**f**) and schematic view (**g**) of Pd/Ni/Au (10/10/30 nm) annealed in RTA in N_2_ + O_2_ flow at 530 °C.

Like the preceding case, we can observe the decomposition of GaN. TEM and EDS data provide evidence of a certain degree of separation between Pd and GaN by Au and Ni (Figure 10). Towards the bottom of the contact, spheroidal islands exist with a thickness of around 15–20 nm, displaying irregular sizes. These islands primarily consist of Pd atoms and diffused Ga. The GaPd alloy is subsequently covered by Ni–Au mixture and/or AuPd species.

EDS map data illustrate a uniform distribution of Pd in the upper Au layer. EDX data indicate that the diffusion of Pd atoms into the Au layer is more noticeable, and Pd distribution in this layer appears more even compared to the sample annealed in N_2_. The thickness of the AuPd layer varies between 30–40 nm. The differing shades within the PdGa or AuPd layers correspond to the variations in composition within these layers.

Despite the decomposition of GaN, no GaxOy and NxOy phases are formed after annealing in an oxygen-containing atmosphere.

Two novel interfaces were formed: AuPd/NiO and GaN/Au/Ni/PdGa. The annealed metallization primarily consists of PdGa, with a smaller region featuring gold and nickel at the lower end, Au and AuPd in the middle and NiO at the top (Figure 10g).

It is important to note that the contact structure annealed in an atmosphere containing O_2_ undergoes species mixing, but void generation was not observed. The absence of voids in the metallization annealed in an oxidized atmosphere prevents uneven current injection and the development of hotspots within the contact region.

**Figure 11:**
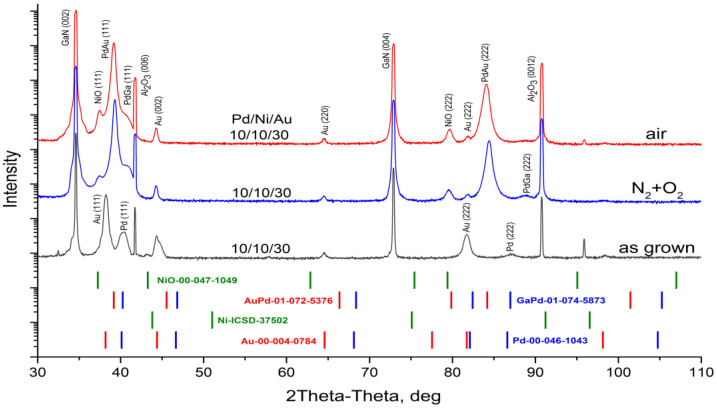
XRD profiles for investigated as-grown sample after annealing by RTA in N_2_ + O_2_ flow.

EDX data were supported by XRD analysis (Figure 11).

XRD findings confirm that after annealing in a N_2_ + O_2_ atmosphere, phases of NiO, PdGa and PdAu appear. The intensity of the Ni (111) reflection disappears, indicating either that too little of the metal is present to be detected by XRD or that the pure Ni is completely dissolved. Low intensities of Au (002) and Pd (111) reflections indicate that these layers have been almost completely consumed by interfacial and interlayer reactions.

Due to its catalytic activity, Ni can prompt the desorption of hydrogen from the Mg–H complex within the p-GaN substrate, a process that might not have occurred during post-growth Mg activation [20]. This desorption could subsequently decrease the electrical passivation of the acceptors, leading to a reduction in the Schottky barrier width and, consequently, a decline in contact resistivity. Another supporting factor for this role of Ni is the absence of Ni_x_Ga_y_ intermetallic compounds at the interface, which generally exhibit a low work function and have the potential to elevate the Schottky barrier height.

Finally, we also conducted contact metallization annealing using an open-air resistive furnace. The distinctions between RTA in simulated air and furnace annealing in a laboratory atmosphere lie in the subtle variations in the temperature–time profile and the introduction of water vapor into the laboratory atmosphere. The outcomes of TLM characterization suggest a slightly lower specific contact resistance for the metallization annealed in the open furnace (Table 2). While there is no definitive understanding of this, one can speculate about the potential role of water vapor in facilitating hydrogen removal.

### 3.4. Three Methods of Contact Preparation—Summary

Microscopic studies have indicated a significant correlation between the contact structure’s morphology and the atmosphere’s composition during annealing. As per the results, the nature of the gas atmosphere during heat treatment notably impacts Ga out-diffusion, intermixing of metals and void generation. Among the most notable effects are:

- Extensive gallium diffusion into the palladium layer and oxidation of nickel when the metallization undergoes annealing in an O_2_-containing atmosphere;
- Void formation in a nitrogen-containing atmosphere;
- Existence of Au–Ni mixture that acts as an intermediate layer between the GaN and metal structure.

Table 2 presents the recorded contact resistances for various annealing methods.

The specific contact resistivity numbers presented for non-ohmic contacts (as-deposited and annealed in N_2_) were calculated as averages. This is due to the challenges in precise measurement using the classical TLM method [26].

The ohmic contact characteristics can be linked to both the arrangement of the metallization structure and the interactions occurring at the metal–p-GaN interface. Another plausible factor contributing to the enhancement of contact resistivity is the potential increase in contact area and adhesion. Thermal treatment has the capacity to induce interface roughening through interfacial reactions, thereby promoting better adhesion. The diminished surface uniformity might arise from variations in material thermodynamic properties, possibly compounded by Pd disrupting some interfacial contamination. This disruption could facilitate enhanced current flow across the interface. The elevated contact resistance compared to that reported in [27] suggests that, in the context of annealing with RTA in N_2_ + O_2_, a shorter treatment time might be more effective in reducing the specific contact resistance.

The reduced contact resistance observed in samples annealed in a furnace can be rationalized by a tighter contact structure. Annealing in an air environment encourages the formation of a more cohesive film, owing to the substantial melting impact of initially deposited sponge-like metals. This effectively prevents the out-diffusion of N_2_ and void formation. Additionally, this process governs the suboptimal wettability observed on the GaN substrate.

The outcomes from RTA annealing in artificial air and oven annealing in air are encouraging and have been employed as metallization techniques in laser diodes.

### 3.5. Influence of the Pd Thickness on the Contact Characteristics

**Figure 12:**
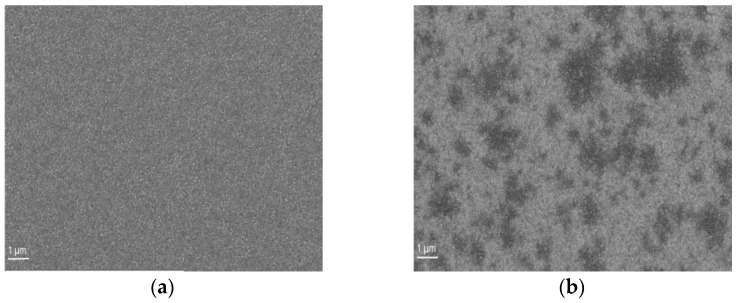
SEM data of Pd/Ni/Au (90/10/30 nm) after annealing by (**a**) oven in N_2_ + O_2_ + H_2_O and (**b**) RTA in N_2_ + O_2_.

To achieve a more uniform metal system, we enhanced the thickness of the Pd layer to mitigate the potential migration of Ni and Au to the GaN interface. The samples were subjected to annealing using both an oven in air and RTA in a N_2_ + O_2_ composition to study the impact of the thermal treatment method. Contacts based on thick Pd layers exhibit distinct morphologies after annealing in an oxidizing environment (Figure 12). However, the specifics are contingent on the type of annealing apparatus employed, such as RTA versus oven.

In Figure 12a, we observe the metallization with a thick (90 nm) Pd layer after annealing in artificial air. Evidently, we note the emergence of inhomogeneities in the metal layers akin to those described in the preceding sections. Figure 12b demonstrates that the external appearance of the contact in the SEM images is significantly smoother for specimens annealed in the oven.

**Figure 13:**
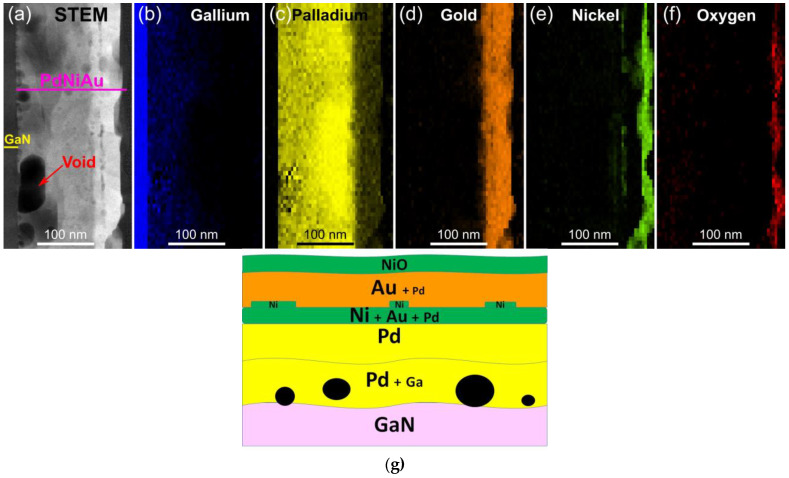
EDX maps of Pd/Ni/Au (90/10/30 nm) after annealing by oven in N_2_ + O_2_ + H_2_O (**a**–**f**) and scheme of Pd/Ni/Au (90/10/30 nm) composition (**g**).

Obtained STEM images revealing thick Pd-based contacts annealed in an oxygen-containing atmosphere surprisingly exhibit the presence of voids at the metal–GaN interface (Figure 13a,g).

According to EDX data, it is observable that the heating of contacts with a thicker Pd layer leads to a more extensive diffusion of Ni through the upper Au layer to the uppermost section of the metallization, where it subsequently undergoes oxidation (Figure 13b–f).

The thickness of the Pd layer is increased to 130 nm, with approximately 50% of the layer near the interface containing Ga atoms. The varying shades of the Pd layer indicate the diverse composition of the solution, a fact corroborated by the fluctuations in the measured interplanar distances (refer to Table 3). For the PdGa alloy, the latter increases from 2.18 to 2.20 Å with decreasing Ga concentration in the lower contact layer. According to EDX data, the lower Pd layer encompasses around 12% gallium. Roughly 18% of palladium diffuses into the upper Au layer.

When examining the intricacies of the metal-layer arrangement in both scenarios (annealing in oven and RTA), that is, with pure and uniform Pd layers, the Pd layer is consistently situated in the middle of the structure. However, heating in a N_2_ + O_2_ flow triggers the development of two distinct types of PdGa solutions (Table 3).

In comparison to the PdGa mixture, the formation of the AuPd solution resulted in less alteration in the thickness of the initial metal layer. The upper Au layer is expected to impede the out-diffusion of Ni species to the surface; however, EDX data reveal that the 30 nm Au layer is insufficient in curbing the out-diffusion of Ni.

Towards the upper portion of the metal structure, a blend of Au, Ni and Pd is present, topped by NiO. The sequence of as-deposited Ni and Au is reversed after undergoing thermal treatment. In a broader sense, the migration of Ga and Au into Pd represents the predominant kinetic mechanism for altering the metal–substrate interfacial energy. Notable variations in the composition of the layers were observed for different sample treatments, evident through the lattice parameters (refer to Table 3). These variations are more pronounced in the XRD data, where lattice parameters can be determined with higher precision; this is discussed later on.

It is important to highlight that annealing using RTA leads to more pronounced reconstruction and interaction between the metal and GaN species. The annealed samples exhibit more a disordered and deteriorated microstructure characterized by a higher number of voids compared to the sample subjected to furnace annealing.

**Figure 14:**
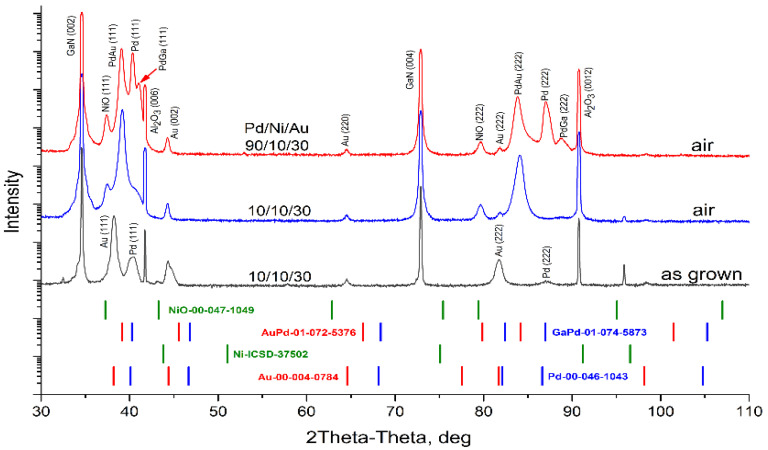
XRD profiles for investigated as-grown sample and after annealing.

XRD analysis was conducted to confirm EDX data (Figure 14). XRD findings suggest that pure Au, Pd and Ni layers are consumed through interfacial and interlayer reactions, which are contingent on the structure and gas atmosphere. It is possible that Pd is entirely dissolved in samples with thin metal layers. Intensity of the Ni (111) reflection disappears which either means that too little of the metal exists to be detected by XRD or that the pure Ni is entirely dissolved. The lower intensity of Au (002) indicates that a small portion of pure Au is still present. Still existing after annealing, the intensity of Pd (111) reflection depends on the as-deposited thickness of Pd and is higher in the case of a thicker layer which indicates the presence of pure Pd in the contact design.

XRD data analysis confirms that after annealing in the air, in both cases of metallization, with thin and thick palladium layers, new phases of NiO, PdGa and PdAu appear. The positions of reflections for these phases vary in XRD profiles, indicating changes in their compositions. The composition assessment results of the PdAu alloy, according to Vegard’s law, are presented in Table 4.

As shown in Table 4, annealing in nitrogen has the strongest effect on the formation of the PdAu phase. In the case of the heating method, treatment in the oven has a lesser impact on the reaction between Au and Pd species. This could be attributed to the gas flow during RTA, which promotes accelerated diffusion of metal species. Considering the variation in Pd concentration in both the PdGa and AuPd mixtures, it can be inferred that the post-reaction products likely exhibit characteristics of a mechanical solution rather than a chemical compound.

In contrast to findings in [6,12] and despite the high diffusion activity of Ni, it does not react with the out-diffused Ga atoms, and Ni–Ga–O phases were not observed. In [9], authors illustrated that the smooth surface of the Pt/Ni/Au contact post-annealing can be attributed to the formation of nickel oxide. In our case, however, annealing in a N_2_ + O_2_ environment results in the roughening of the upper Ni layer, whose thickness varies from 5 to 30 nm.

The formation of NiO is energetically favored by its negative Gibbs free energy (ΔG = −83.7 kJ/mol) at 500 °C [28]. This rapid oxidation process serves as a driving force for the further out-diffusion of Ni atoms until all Ni is converted into the observed continuous NiO surface layer. The presence of a small amount of pure Ni indicates incomplete conditions (time, available oxygen) for complete Ni oxidation. As a diffusion barrier, NiO can enhance the stability of the metallization structure, preventing degradation of the surface and electrical parameters of the contact after annealing [29]. A NiO film covers the top of the metallization, ensuring close contact of Au with the Pd layer and the p-GaN surface [30].

The NiO phase of the oxidized Pd/Ni/Au system remains separate from the p-GaN. Furthermore, the applied temperature is too low for oxide diffusion onto GaN. In our experiments, Au acts as a template for NiO growth due to their quite similar lattice constants (a = 0.408 nm for Au and a = 0.417 nm for NiO) [31]. The epitaxial growth of a NiO structure could generate a single barrier determining carrier scattering through different barriers. Moreover, good species matching eliminates grain boundaries that act as traps and recombination centers for carriers. In fact, more Ni species are oxidized during oven heating, contributing to a lower contact resistivity compared to samples subjected to short-time annealing.

Fast Fourier transform analysis of the metal areas revealed that Pd, Au and NiO species grew in alignment with p-GaN, akin to samples with a thin Pd layer. The orientation relationships between Pd, NiO, Au and p-GaN are akin to those described in [12].

For comparison, Pd/Ni/Au contacts with differing thicknesses of the palladium layer were uniformly annealed in a N_2_ + O_2_ atmosphere composition. Changing the Pd layer thickness has minimal impact on the electrical properties (refer to Table 5). The specific contact resistance slightly diminishes in comparison to contacts with a thin palladium layer. In line with [32], the higher contact resistance of the thin Pd system is attributed to the formation of a larger number of voids.

Experiments indicate that a thick Pd layer effectively serves as a barrier to prevent the diffusion of Au and Ni species to the metal–semiconductor interface. However, increasing the thickness of the Pd layer also leads to a more pronounced decomposition of GaN and the formation of voids.

The small but visible enhancement of the ohmic properties can be attributed to the heightened protection provided by the thicker Pd layer against interfacial reactions involving Ni, Au and the semiconductor. The findings obtained do not align with the suggestion made in [33] that the formation of Pd–Ni–Ga- or Au–Ga-related compounds is necessary to reduce the contact resistance.

The presence of voids diminishes the contact area and results in non-uniform current distribution at the compromised interface. Nevertheless, I–V measurements affirm that even in the presence of voids, the Pd/Ni/Au contact system demonstrates favorable ohmic characteristics.

The fabricated LD (laser diode) structures featuring Pd/Ni/Au metallization were characterized by an operating voltage of approximately 4.1 V, observed at 50 mA or 3.3 kA/cm².

## 4. Conclusions

We investigated Pd/Ni/Au/p-GaN contact systems with varying Pd thickness and subjected them to different heat treatments. As-deposited contacts exhibit non-ohmic behavior and high contact resistance in the order of 7 × 10^−1^ Ωcm².

Heat treatment results in GaN decomposition and the formation of PdGa and PdAu due to interfacial reactions in all samples. The positions of reflections for these phases vary in XRD profiles, indicating changes in their compositions. In the case of the heating method, treatment in the oven has a lesser impact on the reaction between Au and Pd species. This could be attributed to the gas flow during RTA, which promotes accelerated diffusion of metal species. Considering the variation in Pd concentration in both the PdGa and AuPd mixtures, it can be inferred that the post-reaction products likely exhibit characteristics of a mechanical solution rather than a chemical compound.

Annealing of the contact with a 10 nm Pd layer in N_2_ induces gallium out-diffusion, leading to void formation at the semiconductor–metal interface. The contact resistance for N_2_-annealed contacts remains high, comparable to that of the as-deposited metallization.

Annealing metal contacts in an oxidizing atmosphere results in significant restructuring of the three-layer contact, notable gallium migration to the Pd layer and the transformation of Ni into NiO and its subsequent migration to the surface. Opposite to the treatment in the nitrogen atmosphere, voids at the semiconductor–metal interface do not occur. Annealing in an oxidizing atmosphere drastically reduces the contact resistance to a level of 1–3 × 10^−4^ Ωcm², which is an acceptable value for contacts in laser diodes.

In the case of the p-GaN/Pd/Ni/Au (10/10/30 nm) contact structure, neither the method of thermal treatment nor the gas composition influences the separation of semiconductor from metallization by the Au–Ni mixture.

For metal layers with thick palladium layers (90 nm), we observe a metal–semiconductor interface free of Ni and Au, while still maintaining a low contact resistance. This observation raises further questions about the role of oxygen in the annealing atmosphere, with respect to the importance of NiO for these contacts.

It has been demonstrated that Pd, Au and NiO species with an orientation of [111] grow in parallel to p-GaN [0001].

To summarize, annealing in an oxidizing atmosphere leads to GaN decomposition and gallium out-diffusion, extensive species inter-diffusion and mixing and Ni oxidation. The generation of voids depends on the thickness of the Pd layer and the gas atmosphere. Meanwhile, treatment in a gas flow using RTA has a stronger impact on the mixing of Au and Pd. The reduction in the contact area at the interface due to void formation does not necessarily determine an ohmic characteristic of contacts. However, it might limit the reliability of the fabricated device.

## Figures and Tables

**Table 1:** Parameters * of metals and alloys used to produce the ohmic contacts to p-GaN; data taken from [13].

Metal	W [eV]	Melting Point, *T* [°C]	*a*_0_ [nm]	α [10^–6^/K^–1^]	ρ [10^−6^ Ωcm]
Pd	4.80	1550	0.389	11.75	10.8
Ni	4.50	1453	0.352	13.20	6.8
Au	4.25	1063	0.408	14.00	2.3

* W—work function of the metal, *a*—lattice constant, α—thermal expansion coefficient, ρ—resistivity.

**Table 2:** Comparison of the dependences of gas condition and method of heating on the contact resistance.

Thermal Treatment	Specific Contact Resistance (ρ_c_), [Ωcm^2^]	Contact Characteristic
As-deposited	7.3 × 10^−1^	non-ohmic
Oven, air	1.4 × 10^−4^	ohmic
RTA, N_2_ + O_2_	4.0 × 10^−4^	ohmic
RTA, N_2_	3.7 × 10^−1^	non-ohmic

**Table 3:** Interplanar distances for indicated lattice planes after heating in oxidizing ambience by RTA and oven, calculated from STEM data.

RTA, N_2_ + O_2_	Oven, N_2_ + O_2_ + H_2_O
NiO (111) = 2.42 ± 0.01 Å	NiO (111) = 2.41 ± 0.01 Å
AuPd (111) = 2.32 ± 0.01 Å	AuPd (111) = 2.31 ± 0.01 Å
Pd (111) = 2.26 ± 0.01 Å	Pd (111) = 2.24 ± 0.01 Å
PdGa (111) = 2.20 ± 0.01 Å	PdGa (111) = 2.20 ± 0.01 Å
PdGa (111) = 2.18 ± 0.01 Å	GaN (0002) = 2.593 Å
GaN (0002) = 2.593 Å	NiO (111) = 2.41 ± 0.01 Å

**Table 4:** Estimated Pd content in PdAu alloys according to XRD data.

Sample (Pd/Ni/Au)	Annealing Atmosphere	X(%) in Pd_x_Au_1-x_ Alloy
I: 10/10/30 nm	air	51 (±2)
II: 10/10/30 nm	N_2_	67 (±2)
III: 10/10/30 nm	N_2_ + O_2_	58 (±2)
IV: 90/10/30 nm	air	47 (±2)

**Table 5:** Dependence of the contact resistivity on the Pd thickness.

Thickness, [nm]	Specific Contact Resistance, (ρ_c_) [10^−4^ Ωcm^2^]
10/10/30	2.52
30/10/30	2.28
60/10/30	1.74
90/10/30	1.68

## Data Availability

The data presented in this study are available on request from the corresponding author. The data are not publicly available due to privacy reasons..
